# Editorial: Relationship Between Cardiovascular Disease and Other Chronic Conditions

**DOI:** 10.3389/fcvm.2022.875551

**Published:** 2022-04-25

**Authors:** Cristina Vassalle, Junjie Xiao, Laura Sabatino

**Affiliations:** ^1^Fondazione National Council of Researches-Regione Toscana Gabriele Monasterio, Pisa, Italy; ^2^Shanghai Engineering Research Center of Organ Repair, School of Life Science, Shanghai University, Shanghai, China; ^3^Institute of Clinical Physiology, National Council of Researches, Pisa, Italy

**Keywords:** cardiovascular disease, cardiovascular prevention, CV risk assessment, CV prediction, CV outcomes

## Biomarkers and Risk Factors

Cardiovascular disease (CVD) remains the most common cause of mortality and comorbidity all over the world.^1^ Risk stratification plays an important role in the prevention of the onset of cardiovascular disease and the development of its complications. As CVD is multifactorial, several associated risk factors have long been identified (e.g., smoking habit, improper diet, low physical activity, hypertension, hypercholesterolemia, diabetes) (1). The key question is whether we have enough biomarkers to better stratify CV risk. Although the CV burden is generally featured by these traditional risk factors, the importance of non-traditional biomarkers may be critical to fill the gap related to the so-called “residual risk.” In fact, CV load cannot be entirely explained by traditional cardiovascular risk factors, as many patients at risk for adverse prognosis do not present any of these traditional determinants (2). Such evidence suggests the involvement of possible overlooked non-traditional biomarkers that may play an important role in the pathogenesis and development of CVD (2). Thus, there is an increasing interest for additional biomarkers and risk factors providing potential new tools against CVD onset and progression. Discovery of such factors and, most important, of their mechanism of action, may help in the development of advanced care strategies aiming to the reduction of cardiovascular harmful impact. For example, between biochemical factors, beside the widely employed troponins (ischemic damage) and natriuretic peptides (cardiac stress and dysfunction), it would be important to better clarify the role of already known biomarkers in this new context, such as vitamin D well known for bone health, or other new proposed parameters (e.g., oxidative stress biomarkers, hemoglobin, galectin-3-fibrosis, cystatin-renal dysfunction, cytokines and neutrophils/lymphocytes-inflammation, d-dimer-coagulation). However, despite the best efforts, so far, no reliable applicable additive biomarkers have been identified.

A list of all contributions to this special issue is reported in Table 1.

**Table 1:** List of manuscripts included in the special issue.

**Topic**	**Publication type**	**Title**	**Authors**	**Key words**
**Biomarkers and risk factors**	SR	Long-Term Exposure to Ambient Air Pollution and Myocardial Infarction: A Systematic Review and Meta-Analysis	Zou L et al.	PM10; PM2.5 (AQI); air pollution; meta-analysis; myocardial infarction; particulate matter
	SR	Effect of Uric Acid-Lowering Agents on Patients With Heart Failure: A Systematic Review and Meta-Analysis of Randomised Controlled Trials	Xu H et al.	uric acid, hyperuricemia (HUA), heart failure, left ventricular ejection fraction, six minute walk test, B type natriuretic peptide, mortality
	SR	Hypertension in Children and Adolescents: A Position Statement From a Panel of Multidisciplinary Experts Coordinated by the French Society of Hypertension	Bouhanick B et al.	French position statement; adolescents; children; high blood pressure; hypertension
	OR	Increased Neutrophil Respiratory Burst Predicts the Risk of Coronary Artery Lesion in Kawasaki Disease	Hu J et al.	Kawasaki disease, coronary artery lesion, neutrophil, flow cytometry, brain natriuretic peptide
	OR	Prognostic Value of N-Terminal Pro-B-Type Natriuretic Peptide and Glomerular Filtration Rate in Patients With Acute Heart Failure	Wang K et al.	acute heart failure (AHF), N-terminal pro-B-type natriuretic peptide (NT-proBNP), glomerular filtration rate (GFR), outcomes, prognosis
	OR	Prediction Factors of 6-Month Poor Prognosis in Acute Myocardial Infarction Patients	Yao J et al.	acute myocardial infarction, prognosis, death, readmission, biomarker
	OR	Vitamin D Deficiency and Vasovagal Syncope in Children and Adolescents	Zhang Q et al.	children and adolescents, vasovagal syncope, vitamin D, heart rate variability, autonomic nervous function
	OR	Neutrophil to Lymphocyte Ratio Is Increased and Associated With Left Ventricular Diastolic Function in Newly Diagnosed Essential Hypertension Children	Hou M et al.	hypertension, children, neutrophil-lymphocyte ratio, left ventricular hypertrophy, left ventricular diastolic function
	OR	Sex-Specific Associations of Risks and Cardiac Structure and Function With Microalbumin/Creatinine Ratio in Diastolic Heart Failure	Wei F-F et al.	heart failiure, chronic kindney disease, echocardiography, microalbuminuria, risk stratification, gender
	OR	Cystatin C-Based Renal Function in Predicting the Long-Term Outcomes of Chronic Total Occlusion After Percutaneous Coronary Intervention	Li B et al.	cystatin C, creatinine, estimated glomerular filtration rate, chronic total occlusion, all-cause mortality, cardiac death
	OR	Role of Neutrophil-Derived S100B in Acute Myocardial Infarction Patients From the Han Chinese Population	Cheng M et al.	S100B; acute myocardial infarction; genotype; plasma biomarkers; thrombosis
	OR	Cardio-Ankle Vascular Index Reflects Impaired Exercise Capacity and Predicts Adverse Prognosis in Patients With Heart Failure	Watanabe K et al.	cardio-ankle vascular index, arterial stiffness, cardiopulmonary exercise testing, heart failure, prognosis
	OR	Cardiovascular Risk Awareness and Calculated 10-Year Risk Among Female Employees at Taibah University 2019	Qasem Surrati AM et al.	cardiovascular disease, knowledge, awareness, risk factors, Madinah-KSA, calculated 10 year risk
	OR	Effect of Air Quality on the Risk of Emergency Room Visits in Patients With Atrial Fibrillation	Liang B et al.	PM2.5, atrial fibrillation, risk of emergency room visit, monsoon climate region, patients
	OR	Prevalence and Predictors of Left Ventricular Diastolic Dysfunction in Malaysian Patients With Type 2 Diabetes Mellitus Without Prior Known Cardiovascular Disease	Han Chee KH et al.	diastolic dysfunction, diabetes mellitus, left ventricular dysfunction, prevalence, Asian
	OR	Longitudinal Effect of Hemoglobin Concentration With Incident Ischemic Heart Disease According to Hepatic Steatosis Status Among Koreans	Jung DH et al.	hemoglobin, hepatic steatosis, cohort study, ischemic heart disease, risk factor, extrahepatic complications
	OR	Association Between C-Peptide Level and Subclinical Myocardial Injury	Chen Z et al.	C-peptide, subclinical cardiac injury, NHANES III, association, cross sectional study
	OR	UPLC-MS-Based Serum Metabolomics Reveals Potential Biomarkers of Ang II-Induced Hypertension in Mice	Yang S et al.	hypertension, LC-MS, angiotensin II, metabolomics, serum metabolites, biomarkers, mice
	OR	Correlation Analysis of Anti-Cardiolipin Antibody/D Dimer/C-Reactive Protein and Coronary Artery Lesions/Multiple-Organ Damage in Children With Kawasaki Disease	Xu Y-M et al.	anticardiolipin antibody (ACA), D dimer, C reactive protein (CRP), coronary artery lesions (CALs), multiple organ damage, Kawasaki disease (KD), children
	OR	Hyperuricemia Predicts Adverse Outcomes After Myocardial Infarction With Non-obstructive Coronary Arteries	Mohammed A-Q et al.	myocardial infarction, MINOCA, serum uric acid, hyperuricemia, outcome
	OR	Relative contribution of plasma homocysteine levels vs. traditional risk factors to first stroke: a nested case-control study in rural China	Zhou F et al.	homocysteine, systolic blood pressure, first stroke, ischemic stroke, population attributable risk
	CS	A Chromosomal Inversion of 46XX, inv (6) (p21.3p23) Connects to Congenital Heart Defects	Cheng L et al.	congenital heart disease, ventricular septal defect, chromosomal rearrangement, human chromosome 6, proband
**CVD and other conditions**	R	Impact of Increased Oxidative Stress on Cardiovascular Diseases in Women With Polycystic Ovary Syndrome	DuicăF et al.	polycystic ovary syndrome, cardiovascular disease, oxidative stress, C-reactive protein, homocysteine, miRNA
	R	Association Between Periodontal Disease and Atherosclerotic Cardiovascular Diseases: Revisited	Zardawi F et al.	periodontal therapy, relation, periodontal disease, cardiovascular diseases, atherosclerosis
	R	Metabolism and Chronic Inflammation: The Links Between Chronic Heart Failure and Comorbidities	Li Z et al.	heart failure, comorbidities, metabolism, chronic inflammation, reactive oxygen species, mitochondria
	R	Relationship Between Sarcopenia and Cardiovascular Diseases in the Elderly: An Overview	He N et al.	sarcopenia, cardiovascular diseases, elderly people, comorbidity, aging
	OR	Gallbladder Polyps Increase the Risk of Ischaemic Heart Disease Among Korean Adults	Lee Y-J et al.	gallbladder, polyps, coronary disease, comorbidity, cohort study
	OR	Risk Factors of Atrial Arrhythmia in Patients With Liver Cirrhosis: A Retrospective Study	Lu X et al.	age; ascites; atrial arrhythmia; liver cirrhosis; risk factor
	OR	Clinical characteristics of cryoglobulinemia with cardiac involvement in a single center	He K et al.	cryoglobulinemia, cardiac involvement, clinical characteristics, treatment outcome, retrospective study
	CS	Chronic Thromboembolic Pulmonary Hypertension in a Child With Sickle Cell Disease	Spencer R et al.	CTEPH—chronic thromboembolic pulmonary hypertension; hematology; pediatric cardiology; pulmonary hypertension; sickle cell disease
	CS	Right Atrial Thrombus in a COVID-19 Child Treated Through Cardiac Surgery	Bigdelian H et al.	COVID-19, cardiac surgery, pediatric, thrombus—echocardiography, fever
	CS	Massive Right Atrial Thrombosis: Are You Brave Enough to Start Anticoagulation? A Case Report	Bergonti M et al.	right atrial thrombosis, pulmonary thromboembolism, thrombus, cardio-oncology, coagulation, right atrium mass
	BRR	Gout Is Prevalent but Under-Registered Among Patients With Cardiovascular Events: A Field Study	Calabuig I et al.	gout, prevalence, cardiovascular event, cardiovascular disease, urate lowering therapy
	O	Chronic Secondary Cardiorenal Syndrome: The Sixth Innovative Subtype	Zhang Y et al.	biomarker; chronic co-impairment; chronic secondary cardio renal syndrome; fibrosis; new classification; type 6 cardio renal syndrome
**Prevention, diagnosis, treatment**	OR	Timing of Maximal Weight Reduction Following Bariatric Surgery: A Study in Chinese Patients	Xu T et al.	bariatric surgery, Chinese patients, weight reduction, trend, follow up
	OR	Association Between Aspirin Use and Decreased Risk of Pneumonia in Patients With Cardio-Cerebra-Vascular Ischemic Disease: A Population-Based Cohort Study	Chen Y-C et al.	aspirin, pneumonia, risk, database, cardio-cerebra-vascular ischemic diseases
	OR	The Association Between Metformin Treatment and Outcomes in Type 2 Diabetes Mellitus Patients With Heart Failure With Preserved Ejection Fraction: A Retrospective Study	Wang J et al.	heart failure with preserved ejection fraction; metformin; mortality; survival analysis (source: MeSH NLM); type 2 diabetes mellitus—exenatide
	OR	The Complementary Relationship Between Echocardiography and Multi-Slice Spiral CT Coronary Angiography in the Diagnosis of Coronary Artery Thrombosis in Children With Kawasaki Disease	Xu Y-M et al.	children, Kawasaki disease, coronary artery lesion, thrombosis, echocardiography, CTCA

*SR, Systematic Review; OR, Original Research; CR, Case Report; R, Review; BRR, Brief Research Report; O, Opinion*.

Majority of the contributions in this special issue explore the significance of different biomarkers and risk factors in the various CVD manifestations. Among the 21 manuscripts dealing with this issue, we hereby report a few representative examples:

### Inflammation

Regarding inflammation, Hou et al. investigated the “neutrophil to lymphocyte ratio (NLR),” which is a novel inflammatory biomarker, calculated from old well known hemochrome parameters, association with CVD in children with newly diagnosed essential hypertension. Results obtained suggested that a high NLR might be a potential indicator of increased risk of the development of hypertension and LV diastolic dysfunction in children.

In another article, Hu et al. explored the relationship between neutrophil respiratory burst and coronary artery lesions (CAL), suggesting this biomarker as significant in the pathogenesis of CAL and CAL prediction of Kawasaki disease in children.

### Gender/Sex Related Differences

Till now, CVD is perceived as a condition essentially regarding the male gender, although CV events account for the main cause of mortality and morbidity in postmenopausal women (3). Obviously, if it is clear that many aspects of CVD are similar in male and female patients, there are significant differences as well (4, 5). Cellular and molecular mechanisms and clinical manifestations of CVD in women are far from being fully understood. Advances in this field are essential to improve CVD pathophysiological knowledge as well as diagnostic and clinical strategies in women, in order to develop specific female-based algorithms. In this special issue, in particular, two studies addressed different aspects of gender-related characteristics. Wei et al. investigate the relationship between sex-specific associations of adverse health outcomes, left ventricular structure/function and microalbuminuria in patients with heart failure with preserved ejection fraction (HFpEF). The authors found that microalbumin/creatinine ratio (ACR) was significantly associated with LV diastolic function, hospitalization, and myocardial infarction in men, while ACR was associated with mortality in women. The interactions of sex with ACR were significant in heart failure. Moreover, the article of Surrati et al. deals with the important topic of awareness of CV risk. The study was conducted on Saudi Arabia University female employers. Authors reported limited knowledge and awareness of CVD risk, which evidenced the pressing need of educational interventions to enhance the awareness of CVD risk factors and prevention in the female population.

### Arterial Stiffness

Although circulating biochemical markers are extensively studied, other physiological parameters can be effective (6). It is the case of the cardio-ankle vascular index, measured in the study of Watanabe et al. as an indicator of arterial stiffness, which resulted independently associated with impaired exercise capacity and adverse prognosis in HF patients.

### Environmental Pollution

It should not be forgotten the effect of the environment on CVD, a risk factor completely neglected in clinical practice. Interestingly, Zou et al. faced the complex relationship between pollution and cardiovascular disease, reporting the latest evidence on this topic. In their systematic review and meta-analysis including exclusively cohort studies, the authors evidenced the relationship between PM2.5 and PM10 and the risk of myocardial infarction. Sensitivity analyses confirmed and even reinforced these findings. Subgroup analyses by geographical area and year of publication did not show any statistically significant difference in results. Moreover, the study of Liang et al. evidenced as short- and medium-term exposure to PM2.5 significantly increased the risk of emergency room visits in atrial fibrillation patients, suggesting the importance of air quality and providing a rationale to implement actions for reducing CVD risk in the population.

### Multi-Marker Approach

Combining more biomarkers in a multi-marker approach capturing different aspects of CVD (e.g., ischemia, necrosis, thrombosis, inflammation, and fibrosis), may increase the diagnostic and prognostic capacity (7, 8). In this context, Yao et al. investigated the prediction factors of poor prognosis (mortality and/or readmission) after acute myocardial infarction (AMI) during a 6-month follow-up and suggested the multi-biomarker approach using Killip classification 2–4 and myoglobin or creatinine effective for 6-month prognosis prediction in AMI patients. Moreover, Wang et al. investigated the relationship between N-terminal pro-B-type natriuretic peptide (NT-proBNP), Glomerular Filtration Rate (GFR), and outcomes in patients hospitalized with acute heart failure (AHF). The authors found that the risk of death of patients with NT-proBNP>2,137 pg/ml and GFR<61.7 ml/(min·1.73 m^2^) was significantly higher, and suggested that the combination of GFR and NT-proBNP improved the predictive value for the long-term prognosis of AHF patients.

### Genetics, Chromosomal Abnormality, Metabolomics

The study of the genetic components may offer important advances in the pathophysiological aspects of CVD, in the effort of developing more efficient predictive tests for those at high risk (9). Genetic biomarkers are already present at birth and, thus, risk prediction can be evaluated before CV risk factors' onset and development, in a primordial prevention strategy. Accordingly, Cheng et al. investigated the role of neutrophil-derived S100B genetic variants in atherosclerosis progression of acute myocardial infarction (AMI), evidencing how the S100B rs9722 AA homozygous might promote the development of AMI.

Interestingly, Cheng et al. showed that ventricular septal defect (VSD) is closely related to chromosomal aneuploidies by reporting a pedigree with VSD associated with a balanced paracentric inversion of chromosome 6, inv (5) (p21.3p23). This evidence might represent a new genetic etiology for VSD.

New “omic” fields (e.g., metabolomics) are also emerging, rendering reasonable phenotype identification of patients on the basis of biomarker cluster analysis (where multiple co-occurring pathological factors can simultaneously be found in a single clustering) clinically useful in the next future time (10). In this context, the study from Yang et al. suggested that non-targeted metabolomics could evidence biochemical pathways associated with Ang II-induced hypertension in an experimental model. Available data in this field may improve knowledge of systemic metabolic response to sustained release of Ang II, providing a new panel of biomarkers that may be helpful to predict blood pressure changes in the early stages of hypertension.

## CVD and Other Conditions

Until now, the traditional medical approach to diseases has been characterized by a point of view generally focused to diagnose, and treat pathological conditions “*per se*.” Nonetheless, there is now increasingly awareness that diseases apparently independent, instead may share many risk factors and common critical pathophysiological pathways. Twelve articles in this special issue provide evidence on how each organ/system, including CV system, is not a solitary and independent entity, but is part of a whole, interacting within a complex network with other organs (11). This is an important issue, and we need to broaden our knowledge on the differences/similarities between diseases, often considered and managed as separate entities in the common clinical practice. In fact, many pathological conditions, traditionally considered unrelated, emerge as interactive with the cardiovascular system, able to evoke similar different systemic responses, and share underlying cellular pathways and biomarkers. Accordingly, several risk factors, traditionally classified as relevant for the CVD onset and progression (e.g., diet, physical inactivity, hypertension, diabetes), result to be significant in the development of other pathological states. In particular, chronic inflammation and oxidative stress represent commonly underlying aspects in the pathogenesis and progression of different diseases, although additional overlapping mechanisms and further biochemical pathways may have other acting roles. Please, refer to table and find some example below:

Dealing with the interaction between diseases, Bigdelian et al. reported an interesting case discussing the presence of a right atrial vegetation in an 11-year-old child infected by COVID-19. They hypothesized that the etiology is the result of hypercoagulation and acute thrombosis in COVID-19 patients, which raised the issue of possible targeted treatment strategies in COVID-19 patients, to avoid hypercoagulative status and thrombus development.

Calabuig et al. brought their attention to a high gout prevalence among patients admitted for CV events, often undetected and, as such, under suboptimal treatment, despite being a well recognized CV risk factor.

Zardawi et al. drew the attention of dental practitioners and cardiologists on the reciprocal role of periodontal and CVD. This is an intriguing topic for both dentists and cardiologists in recent years, suggesting that CVD patients may benefit from periodontal check, whereas patients with periodontal disease may benefit from periodical CV evaluation. Moreover, authors also discussed available evidence on common factors that may drive the progression of both diseases. Essentially, the main mechanisms involved are the direct invasion of bacteria and inflammation. For what concerns therapy, periodontal treatment was effective in reducing the level of inflammatory biomarkers and improving endothelial function, whereas local application of statin could also improve periodontitis through its anti-inflammatory effect.

Duica et al. reviewed the current literature and provided a new perspective of polycystic ovary syndrome, a reproductive endocrine condition, in the context of key inflammatory and oxidative stress factors and cardiovascular risk. In particular, in women with polycystic ovary syndrome, a link with the increased incidence of CVD was found, highlighting the possibility to apply an antioxidant strategy in this population.

## Prevention, Diagnosis, and Treatment

Critical aspects related to prevention and treatment are faced by four more articles. In the therapeutic field, Chen Y-C et al. provided evidence on long-term low-dose aspirin association with reduced risk of pneumonia in CV patients, suggesting an important role of this drug in the prevention of this critical complication. Instead, Wang et al. evaluated the association between metformin and adverse outcome in T2DM patients with HFpEF, evidencing how metformin in this population was not independently associated with clinical outcomes in patients with T2DM and HFpEF, but resulted related with lower all-cause mortality in the subgroup of patients with poor glycemic control. Moreover, Xu et al. dealt with strengths and limitations of two imaging techniques (transthoracic echocardiography and multi-slice spiral CT coronary angiography) for identifying coronary artery thrombosis in children with Kawasaki disease (KD), evidencing their reciprocal complementarity, and the utility of their combination to improve the diagnosis rate for coronary thrombosis.

## Conclusions

In conclusion, identification of new biomarkers may provide additional pathophysiological information improving biological knowledge of the disease, as well as to help in better risk stratification and identification of new targets of interventional strategies. In this context, the adoption of a multimarker panel may provide significant gain, especially if incorporating biomarkers with a low degree (or even absence) of correlation, as a reflex of different pathophysiological pathways, and, as such, capturing more levels of information.

Nonetheless, more information from non-traditional risk factors and biomarkers is needed before their introduction into the clinical practice, and warnings about possible numerous biases need to be considered (e.g., statistics).

A biomarker is considered helpful when leads to reclassification, with the potential of an incremental gain in subjects at low and intermediate risk, and especially in specific patient groups where traditional biomarkers/scores may be not optimal (e.g., women, elderly population). Thus, the assessment of the incremental value of a proposed biomarker over traditional risk models is a critical step. In this context, advances for “omic” technologies can be considered critical to reveal novel unknown molecular pathways and biomarkers, which may be of importance to characterize a particular disease state when added to traditional algorithms. Although in their developmental phase and still presenting some shortcomings, the integration these technologies in a multi-omics approach and the generation of big data may provide an exceptional opportunity to further understand processes and dynamic interactions underlying human pathophysiology, with a great potential for their relatively rapid diffusion in the routine use (12).

Generally, for the new proposed biomarkers there is a consistent insufficiency of quality controls, primary aspects of assay performance and reliability (e.g., reference material availability, quality assurance programs). Moreover, other different issues must be faced and standardized before biomarker introduction in the clinical practice, for example:

- Preanalytical factors, i.e., all factors generating variability, but controllable or minimized by standardizing the time and condition of sampling, including circadian rhythm and seasonal variation, menstrual cycle, food intake, posture and exercise, sample type (e.g., whole blood, serum, plasma or other specimens), interferences (i.e., lipemia, hemolysis), handling and storage;
- Biological heterogeneity, analytical interferences (e.g., heterophilic antibodies, immunocomplexes);
- Limits of detection (lowest concentration that can be detected) and quantitation (lowest concentration quantitatively measured with accuracy);
- Linearity;
- Reference limits (at least cut-off values);
- Ratio of cost on effectiveness;
- Easiness of use;
- Context of application as:
  - Clinical purpose (screening, diagnosis, prognosis, monitoring, treatment),
  - Condition status (risk or presence or stage of the disease),
  - Target population (e.g., males vs. females, elderly individuals).

The leitmotiv of most manuscripts reported in this issue is that no organ or system, including the CV system, is an “island,” therefore the simultaneous consideration of different conditions, so far treated separately, may be helpful to develop strategies aiming to multi-disease benefits and optimize precision medicine approaches. In this scenario, a close collaboration between cardiologists and other clinical professionals and basic science researchers (e.g., biologists, laboratorists, epidemiologists, radiologists, and physicians in other fields) is desirable.

## Author Contributions

CV: conceptualization and writing—original draft preparation. JX, LS, and CV: review and editing. All authors contributed to the article and approved the submitted version.

## Conflict of Interest

The authors declare that the research was conducted in the absence of any commercial or financial relationships that could be construed as a potential conflict of interest.

## Publisher's Note

All claims expressed in this article are solely those of the authors and do not necessarily represent those of their affiliated organizations, or those of the publisher, the editors and the reviewers. Any product that may be evaluated in this article, or claim that may be made by its manufacturer, is not guaranteed or endorsed by the publisher.

## Footnotes

^1^ https://www.who.int/health-topics/cardiovascular-diseases#tab=tab_1

## References

[1] Voglhuber J Ljubojevic-Holzer S Abdellatif M Sedej S. Targeting cardiovascular risk factors through dietary adaptations and caloric restriction mimetics. Front Nutr. (2021) 8:758058. 10.3389/fnut.2021.758058 34660673 PMC8514725

[2] Traghella I Mastorci F Pepe A Pingitore A Vassalle C. Nontraditional cardiovascular biomarkers and risk factors: rationale and future perspectives. Biomolecules. (2018) 8:40. Erratum in: Biomolecules. (2018). 8(4). 10.3390/biom8040168 29914099 PMC6023023

[3] Mateo-Rodríguez I Danet A Bolívar-Muñoz J Rosell-Ortriz F Garcia-Mochón L Daponte-Codina A. Gender differences, inequalities and biases in the management of acute coronary syndrome. J Healthc Qual Res. (2021) Dec 6:S2603-6479(21)00109-3. 10.1016/j.jhqr.2021.10.010 34887226

[4] Vassalle C Simoncini T Chedraui P Pérez-López FR. Why sex matters: the biological mechanisms of cardiovascular disease. Gynecol Endocrinol. (2012) 28:746–51. 10.3109/09513590.2011.652720 22329808

[5] Connelly PJ Azizi Z Alipour P Delles C Pilote L Raparelli V. The importance of gender to understand sex differences in cardiovascular disease. Can J Cardiol. (2021) 37:699–710. 10.1016/j.cjca.2021.02.005 33592281

[6] Tsai JP Hsu BG. Arterial stiffness: a brief review. Tzu Chi Med J. (2020) 33:115–21. 10.4103/tcmj.tcmj_44_20 33912407 PMC8059465

[7] Vassalle C. New biomarkers and traditional cardiovascular risk scores: any crystal ball for current effective advice and future exact prediction? Clin Chem Lab Med. (2018) 56:1803–5 10.1515/cclm-2018-0490 30016274

[8] Srour B Kaaks R Johnson T Hynes LC Kühn T Katzke VA. Ageing-related markers and risks of cancer and cardiovascular disease: a prospective study in the EPIC-Heidelberg cohort. Eur J Epidemiol. (2021) 22:3. 10.1007/s10654-021-00828-3 34935094 PMC8791871

[9] Hamrefors V. Common genetic risk factors for coronary artery disease: new opportunities for prevention? Clin Physiol Funct Imaging. (2017) 37:243–54. 10.1111/cpf.12289 26278888

[10] Vernon ST Hansen T Kott KA Yang JY O'Sullivan JF Figtree GA. Utilizing state-of-the-art “omics” technology and bioinformatics to identify new biological mechanisms and biomarkers for coronary artery disease. Microcirculation. (2019) 26:e12488. 10.1111/micc.12488 29956866

[11] Vassalle C Iervasi G. Cathepsin K–a classical bone biomarker in cardiovascular disease: the heart is not alone anymore. Atherosclerosis. (2013) 228:36–7. 10.1016/j.atherosclerosis.2013.01.042 23484451

[12] Gaggini M Pingitore A Vassalle C. Plasma Ceramides Pathophysiology, Measurements, Challenges, and Opportunities. Metabolites. (2021) 11:719. 10.3390/metabo11110719 34822377 PMC8622894

